# Evaluation of Constant Thickness Cartilage Models vs. Patient Specific Cartilage Models for an Optimized Computer-Assisted Planning of Periacetabular Osteotomy

**DOI:** 10.1371/journal.pone.0146452

**Published:** 2016-01-05

**Authors:** Li Liu, Timo Michael Ecker, Steffen Schumann, Klaus-Arno Siebenrock, Guoyan Zheng

**Affiliations:** 1 Institute for Surgical Technology and Biomechanics, University of Bern, CH-3014, Bern, Switzerland; 2 Department of Orthopaedic Surgery, Inselspital, Bern, Switzerland; Emory University School Of Medicine, UNITED STATES

## Abstract

Modern computerized planning tools for periacetabular osteotomy (PAO) use either morphology-based or biomechanics-based methods. The latter relies on estimation of peak contact pressures and contact areas using either patient specific or constant thickness cartilage models. We performed a finite element analysis investigating the optimal reorientation of the acetabulum in PAO surgery based on simulated joint contact pressures and contact areas using patient specific cartilage model. Furthermore we investigated the influences of using patient specific cartilage model or constant thickness cartilage model on the biomechanical simulation results. Ten specimens with hip dysplasia were used in this study. Image data were available from CT arthrography studies. Bone models were reconstructed. Mesh models for the patient specific cartilage were defined and subsequently loaded under previously reported boundary and loading conditions. Peak contact pressures and contact areas were estimated in the original position. Afterwards we used a validated preoperative planning software to change the acetabular inclination by an increment of 5° and measured the lateral center edge angle (LCE) at each reorientation position. The position with the largest contact area and the lowest peak contact pressure was defined as the optimal position. In order to investigate the influence of using patient specific cartilage model or constant thickness cartilage model on the biomechanical simulation results, the same procedure was repeated with the same bone models but with a cartilage mesh of constant thickness. Comparison of the peak contact pressures and the contact areas between these two different cartilage models showed that good correlation between these two cartilage models for peak contact pressures (r = 0.634 ∈ [0.6, 0.8], p < 0.001) and contact areas (r = 0.872 > 0.8, p < 0.001). For both cartilage models, the largest contact areas and the lowest peak pressures were found at the same position. Our study is the first study comparing peak contact pressures and contact areas between patient specific and constant thickness cartilage models during PAO planning. Good correlation for these two models was detected. Computer assisted planning with FE modeling using constant thickness cartilage models might be a promising PAO planning tool when a conventional CT is available.

## Introduction

Periacetabular osteotomy (PAO) is an established surgical intervention for treatment of hip dysplasia and acetabular retroversion [1, 2]. During the procedure, the acetabulum is reoriented in order to optimize the containment of the femoral head and the pressure distribution between acetabulum and femoral head for reduction of the peak contact pressures within the joint. The goal of acetabular reorientation is to restore or to approximate normal acetabular geometry. In order to achieve this, two types of planning strategies have been reported, which can be divided into morphology-based planning methods and biomechanics-based planning methods. Morphology-based planning uses standard geometric parameters, which have shown their importance for quantification of acetabular under- or overcoverage [3]. Several authors have described different morphology-based planning methods which range from simplified two-dimensional planning [4–6] to complex three-dimensional planning applications [7–11]. Other authors presented biomechanics-based planning methods. Different approaches have been presented using for example Discrete Element Analysis (DEA) [12], or the more sophisticated Finite Element Analysis (FEA) [13, 14]. In literature, both constant thickness cartilage models [14] and patient specific cartilage models [15] have been suggested. In the clinical routine, knowledge of patient specific cartilage is rarely available, since special imaging protocol (e.g. CT arthrography or MRI with dGEMRIC, T1rho or T2 mapping) is necessary to retrieve this information. One alternative could be constant thickness cartilage model that is virtually generated from bony surface models derived from conventional CT scans. However differences between these two different cartilage models in planning of PAO using FE simulation have never been investigated. Previously, we have developed a morphology-based 3D planning system for PAO [16]. This system allows for quantification of the hip joint morphology in three dimensions, using geometric parameters such as inclination and anteversion angle, the lateral center edge (LCE) angle and femoral head coverage. It also allows for virtual reorientation of the acetabulum according to these parameters. In the current study, we enhanced this application with an additional biomechanics-based method for estimation of joint contact pressures employing FEA. In this study, we investigated the following research questions:

1. What is the optimal position of the acetabulum based on simulated joint contact pressures using patient specific cartilage models in a FE analysis?
2. Are there significant differences in joint contact pressures between patient specific cartilage model and constant thickness cartilage model in the same hip model?

## Materials and Methods

### System Overview

**Fig 1:**
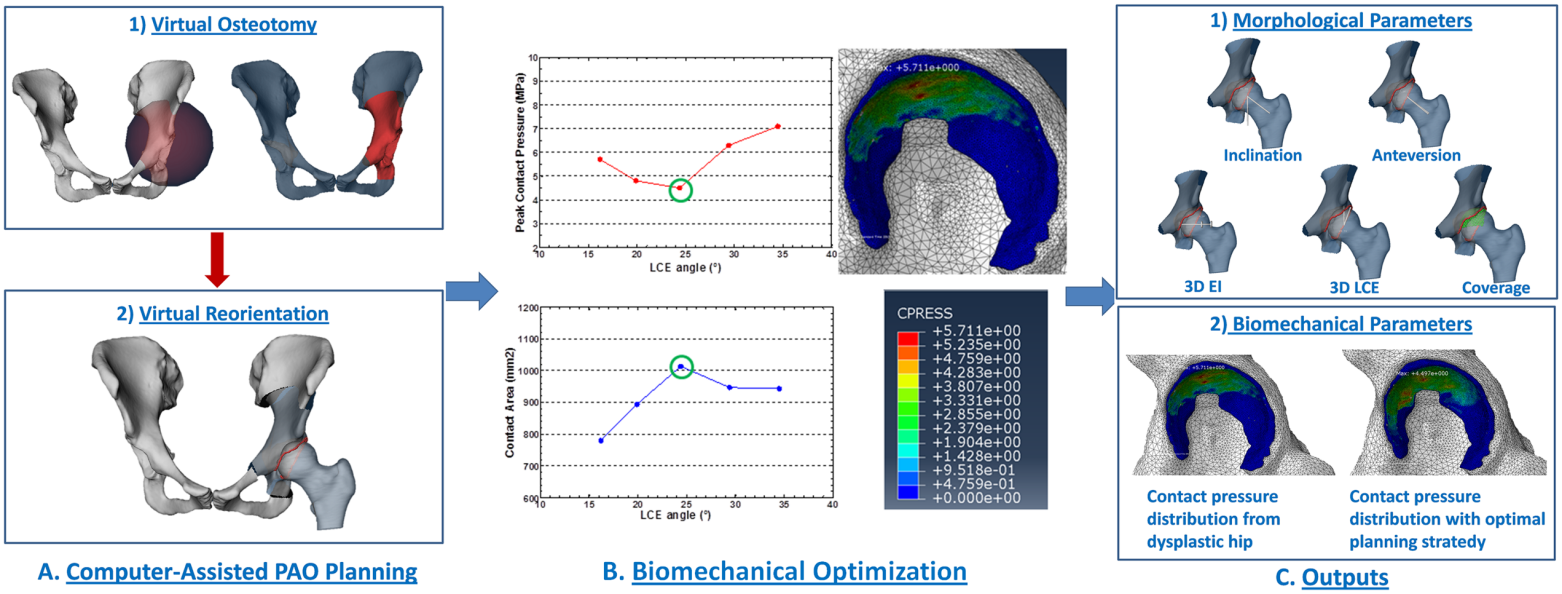
The schematic workflow of computer assisted planning of PAO with biomechanical optimization. (A) Computer assisted morphology based PAO planning. Virtual osteotomy operation is done with a sphere, whose radius and position can be interactively adjusted, and virtual reorientation operation is done by interactively adjusting anteversion and inclination angle of the acetabulum fragment. (B) Biomechanical optimization. (C) the pre-operative planning output.

The computer-assisted planning system for PAO uses 3D surface models of the pelvis and the femur, generated out of DICOM (digital imaging and communication in medicine) data, using a commercially available segmentation program (AMIRA, Visualization Sciences Group, Burlington, MA). The system starts with a morphology based method. Employing fully automated detection of the acetabular rim, parameters such as acetabular version, inclination, LCE angle, femoral head extrusion index (EI), femoral head coverage can be calculated for a computer-assisted diagnosis [16]. Afterwards, the system offers the possibility to perform a virtual osteotomy (Fig 1.A(1)) and reorientation of the acetabular fragment in a stepwise pattern. During the fragment reorientation, acetabular morphological parameters are re-computed in real-time (Fig 1.A(2)) until the desired position is achieved.

Our system is further equipped with a biomechanics-based FE prediction of changes of cartilage contact stresses, which occurs during acetabular reorientation. An optimal position of the acetabulum can be defined, once contact areas in the articulation are maximized, while at the same time peak contact pressures are minimized (Fig 1.(B)).

The respective cartilage model for the biomechanics-based FE prediction is generated from either CT arthrography data (patient specific) or using a virtually generated cartilage with predefined thickness (constant thickness).

### Biomechanical Model of Hip Joint

#### Cartilage models

In literature, both constant thickness cartilage models and patient specific cartilage models have been employed. Zou et al. [14] used a constant thickness model and thus created a cartilage with a predefined thickness of 1.8mm, a value derived from cartilage thickness data from the literature. In contrast Harris et al. [15] introduced a CT arthrography protocol allowing for excellent visualization of patient specific cartilage. DICOM data of dysplastic hip joints, which have been CT scanned using this arthrography protocol were provided by the open source dysplastic hips image data from the Musculoskeletal Research Laboratories, University of Utah [17]. The data provider has obtained IRB approval (University of Utah IRB #10983). We used our morphology-based planning system for calculation of the acetabular morphological parameters [18], verifying true dysplasia (Table 1). We used these datasets in order to retrieve the patient specific cartilage models. The bony anatomy of the same ten specimen was then used to create the constant thickness cartilage models by expanding a constant 1.8mm thickness using 3D dilation operation on the articular surface.

**Table 1:** Acetabular morphological parameters of ten specimen with hip dysplasia.

	**Inclination (°)**	**Anteversion (°)**	**LCE (°)**	**Extrusion Index**	**Coverage (%)**
**#1**	59.7	12.5	17.2	0.33	63.3
**#2**	57.2	10.9	17.1	0.34	62.6
**#3**	58.6	17.1	16.2	0.34	61.8
**#4**	59.0	18.9	19.8	0.31	60.4
**#5**	44.7	16.7	23.1	0.26	69.9
**#6**	59.6	26.7	17.7	0.35	57.4
**#7**	50.5	19.4	23.9	0.25	70.9
**#8**	56.3	23.6	21.0	0.27	66.3
**#9**	60.7	24.7	15.6	0.34	59.3
**#10**	57.4	18.6	18.6	0.30	56.5

#### Mesh Generation

**Fig 2:**
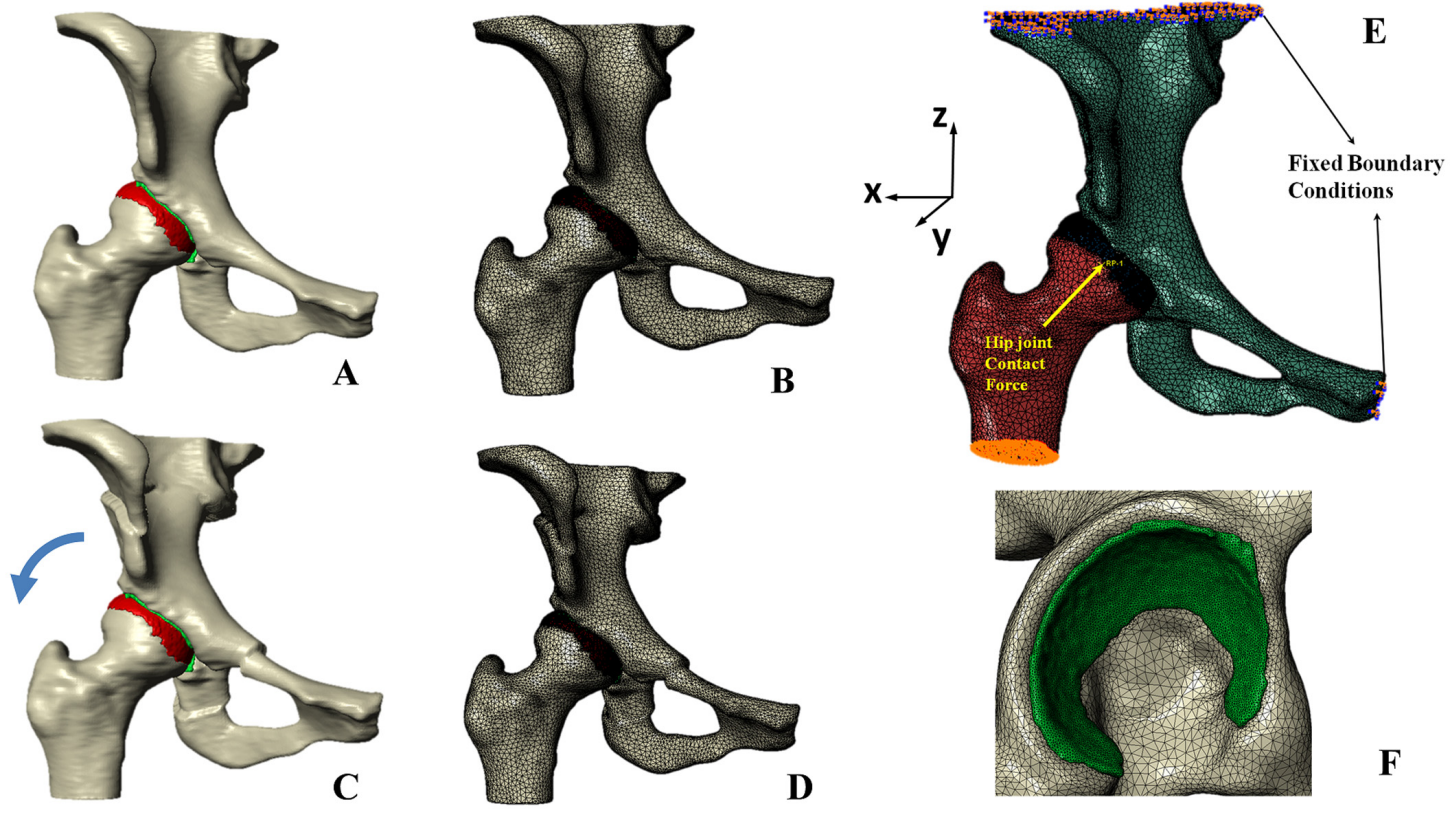
Biomechanical simulation of contact pressure on acetabular cartilage. (A) Surface models of a dysplastic hip; (B) Volume meshes of a dysplastic hip. (C) Surface models for a planned situation after acetabulum fragment reorientation. (D) Volume meshes for the planned situation. (E) Boundary conditions and loading for biomechanical simulation. (F) Coarse meshes for bone models, and refined meshes for cartilages.

Bone and cartilage surface models of the reoriented hip joints were imported into ScanIP software (Simpleware Ltd, Exeter, UK) as shown in Fig 2(A) and 2(C). Surfaces were discretized using tetrahedral elements (Fig 2(B) and 2(D)). Since the primary focus were the joint contact stresses, a finer mesh was employed for the cartilage than for the bone. Refined tetrahedral meshes were constructed for the cartilage models (∼135369 elements for the femoral cartilage model, and ∼92791 elements for the acetabular cartilage model, using the ScanFE module (Simpleware Ltd, Exeter, UK). Cortical bone surfaces were discretized using coarse tetrahedral elements (∼149120 elements for the femoral model, and ∼188526 elements for the pelvic model). Trabecular bone was not included in the models, as it only has a minor effect on the predictions of contact pressure as reported in another study [19].

#### Material property

Acetabular and femoral cartilage were modeled as homogeneous, isotropic, and linearly elastic material with Young’s Modulus E = 15 MPa and Poisson’s ratio *ν* = 0.45 [14]. Cortical bone of pelvis and femur were modeled as homogeneous, isotropic material with elastic modulus E = 17 GPa and Poisson’s ratio *ν* = 0.3 [14].

#### Boundary Conditions and Loading

Tied and sliding contact constraints were used in Abaqus/CAE 6.10 (Dassault Systèmes Simulia Corp, Providence, RI, USA) to define the cartilage-to-bone and cartilage-to-cartilage interfaces, respectively. It has been reported that the friction coefficient between articular cartilage surfaces was very low (0.01–0.02) in the presence of synovial fluid, making it reasonable to neglect eventual frictional shear stresses [15, 20]. The top surface of pelvis and pubic areas were fixed, and the distal end of the femur was constrained to prevent displacement in the body x and y directions while being free in vertical z direction (Fig 2(E)). The center of the femoral head was derived from a least-squares sphere fitting and was selected to be the reference node. The nodes of femoral head surface were constrained by the reference node via kinematic coupling. The fixed boundary condition model was then subjected to a loading condition as published before [21], representing a single leg stance situation with the resultant hip joint contact force acting at the reference node. Following the loading specifications suggested in another previous study [22](Fig 2(E)), the components of joint contact force along 3 axes were given as 195N, 92N, and 1490N, respectively. In order to remove any scaling effect of body weight on the absolute value of the contact pressure, we defined a constant body weight of 650N for all subjects. The resultant force was applied, based on anatomical coordinate system described by Bergmann et al [21], whose local coordinate system was defined with the x axis running between the centers of the femoral heads (positive running from the left femoral head to the right femoral head), the y axis pointing directly anteriorly, and the z axis pointing directly superiorly.

#### Study 1: FE Simulation for biomechanics-based planning of PAO using patient specific cartilage model

In order to find the optimal aceatbular position, the acetabular fragment was now virtually rotated around the y axis (Fig 2(E)) in 5° increments in relation to the anterior pelvic plane (APP). This deemed to imitate a decrease in actabular inclination, as performed during actual PAO surgery (Fig 2(C)). For each increment, the predicted peak contact pressure and total contact area were directly extracted from the output of Abaqus/CAE 6.10. The resulting peak contact pressures and contact areas in the different acetabular positions were then compared and the corresponding LCE angle were measured. Optimal orientation was determined by the position yielding the maximum contact area and the minimum peak contact pressure.

#### Study 2: Evaluation the influences of using different cartilage models on the simulation results

After the peak pressures and contact areas had been simulated using the patient specific cartilage models, the same procedure was performed using the constant thickness cartilage models. Finally, comparison between peak pressures and contact areas between patient specific and constant thickness cartilage models was performed. Linear regression analysis was used to determine associations between the results for peak pressures and contact areas for both cartilage types. Thus, the values for the constant thickness models were the independent variables, whereas the values obtained by the patient specific models represented the dependent variables. Pearson’s correlation coefficient r was interpreted as “poor” below 0.3, “fair” from 0.3 to 0.5, “moderate” from 0.5 to 0.6, “moderately strong” from 0.6 to 0.8, and “very strong” from 0.8 to 1.0. Significance level was defined as p < 0.05.

## Results

**Fig 3:**
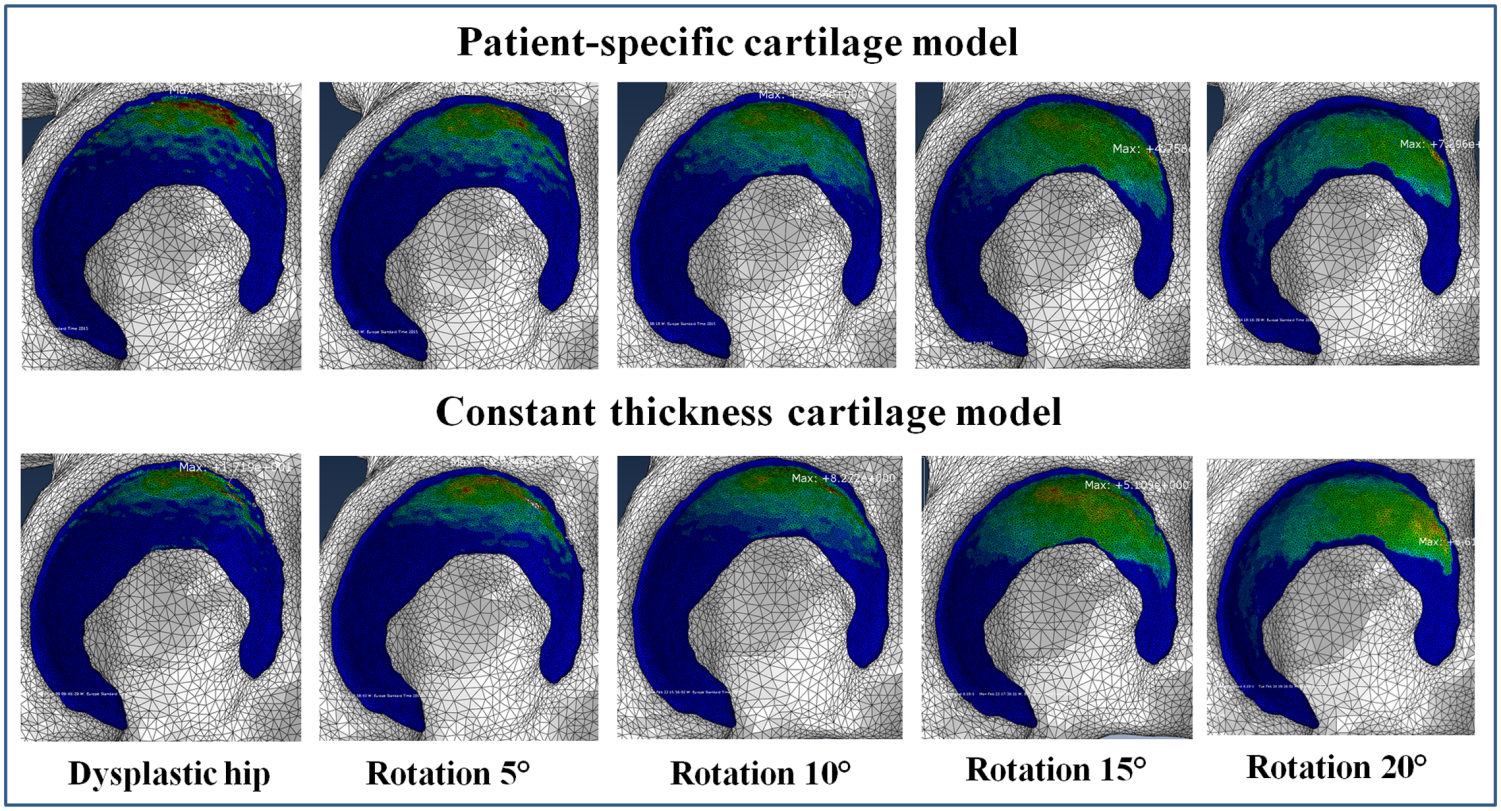
Contact pressure distribution obtained by using two different cartilage models at different acetabular reorientation position.

While the initial contact area in the dysplastic hip was primarily located in an eccentric supero-lateral region of the acetabulum, an increase in LCE angle led to an enlarged and more homogeneously distributed contact area (Fig 3). At the same time, an increase in LCE angle resulted in decreased peak contact pressures. For each specimen, the optimal acetabular fragment reposition was defined as the position with minimum peak contact pressure and maximum contact area (Table 2).

**Table 2:** Acetabular fragment reposition position with peak contact pressures and contact area.

		**R-0°**	**R-5°**	**R-10°**	**R-15°**	**R-20°**
**#1**	**LCE (°)**	17.2	23.0	27.9	**32.9** *	37.9
	**Peak contact pressure (MPa)**	14.1	9.5	7.1	4.8 *	7.3
	**Contact area (mm^2^)**	523	616	778	899 *	860
**#2**	**LCE (°)**	17.1	21.7	**26.8** *	**31.8** *	36.8
	**Peak contact pressure (MPa)**	8.7	6.6	6.3 *	7.0	9.8
	**Contact area (mm^2^)**	625	655	698	741 *	731
**#3**	**LCE (°)**	16.2	19.9	**24.4** *	29.4	34.5
	**Peak contact pressure (MPa)**	5.7	4.8	4.5 *	6.3	7.1
	**Contact area (mm^2^)**	779	894	1013 *	947	943
**#4**	**LCE (°)**	19.8	**23.5** *	28.0	33.0	38.0
	**Peak contact pressure (MPa)**	7.1	6.2 *	8.3	10.2	13.0
	**Contact area (mm^2^)**	1166	1198 *	1096	933	836
**#5**	**LCE (°)**	23.1	27.9	**32.9** *	37.9	43.0
	**Peak contact pressure (MPa)**	5.5	5.2	4.8 *	7.7	9.1
	**Contact area (mm^2^)**	636	769	764 *	587	523
**#6**	**LCE (°)**	17.7	21.5	**26.5** *	**31.6** *	36.6
	**Peak contact pressure (MPa)**	8.6	9.0	8.2 *	8.8	11.1
	**Contact area (mm^2^)**	466	493	517	565 *	468
**#7**	**LCE (°)**	23.9	28.9	**33.9** *	**38.9** *	43.9
	**Peak contact pressure (MPa)**	11.3	9.8	10.0 *	10.0 *	15.0
	**Contact area (mm^2^)**	441	521	586	590 *	485
**#8**	**LCE (°)**	21.0	26.0	31.0	**36.0** *	41.0
	**Peak contact pressure (MPa)**	15.0	10.2	10.8	9.9 *	11.3
	**Contact area (mm^2^)**	469	514	518	530 *	505
**#9**	**LCE (°)**	15.6	19.6	24.6	**29.7** *	34.7
	**Peak contact pressure (MPa)**	10.7	9.3	9.2	7.1 *	8.5
	**Contact area (mm^2^)**	425	381	411	480 *	448
**#10**	**LCE (°)**	18.6	23.0	**28.0** *	32.8	37.8
	**Peak contact pressure (MPa)**	6.6	6.0	4.7 *	9.7	22.5
	**Contact area (mm^2^)**	802	826	951 *	750	699

* represents the position with minimum peak contact pressure and maximum contact area.

**Fig 4:**
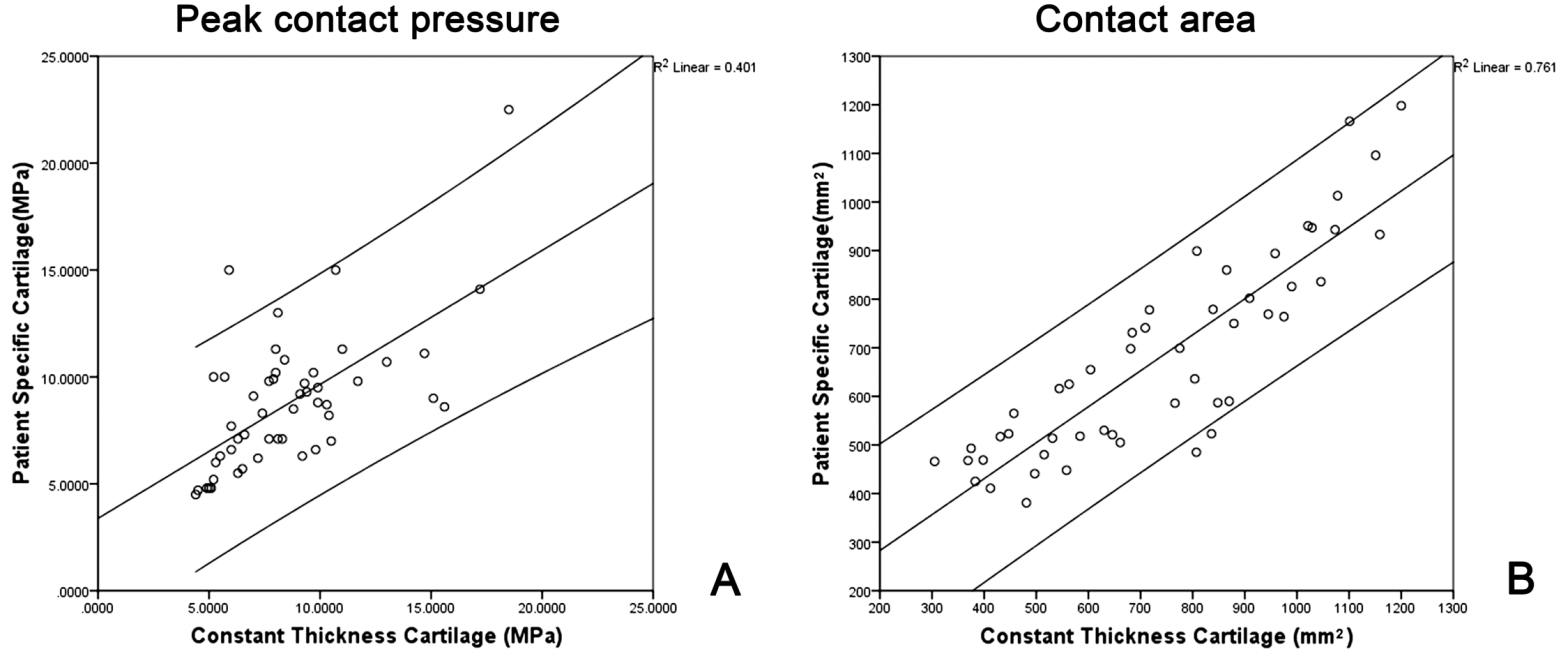
(A) Scatter plot of peak contact pressure obtained by constant thickness cartilage models against those obtained by patient specific cartilage models. (B) Scatter plot of contact area obtained by constant thickness cartilage models against those obtained by patient specific cartilage models.

Comparison of the peak contact pressures and the contact areas between the two different cartilage models showed similar results (Table 3). Regression analysis quantitatively showed that the results obtained by the constant thickness cartilage models had good correlation with those obtained by using the patient specific cartilage models. Specifically, a moderately strong correlation was found between both cartilage models when analyzing peak contact pressures (r = 0.634 ∈ [0.6, 0.8], p < 0.001) (Fig 4) while a very strong correlation was also found when analyzing the contact areas between the two different cartilage models (r = 0.872 > 0.8, p < 0.001) (Fig 4(B)). For both cartilage models, the largest contact areas and the lowest peak pressures were found at the same position (Table 3)

**Table 3:** Acetabular fragment reposition position with peak contact pressures and contact area (Patient specific cartilage model vs. Constant thickness cartilage model).

		**R-0°**	**R-5°**	**R-10°**	**R-15°**	**R-20°**
**#1**	**Patient specific cartilage model**					
	**Peak contact pressure (MPa)**	14.1	9.5	7.1	4.8 *	7.3
	**Contact area (mm^2^)**	523	616	778	899 *	860
	**Constant thickness cartilage model**					
	**Peak contact pressure (MPa)**	17.2	9.9	8.3	5.1 *	6.6
	**Contact area (mm^2^)**	447	544	717	808	865 *
**#2**	**Patient specific cartilage model**					
	**Peak contact pressure (MPa)**	8.7	6.6	6.3 *	7.0	9.8
	**Contact area (mm^2^)**	625	655	698	741 *	731
	**Constant thickness cartilage model**					
	**Peak contact pressure (MPa)**	10.3	9.8	9.2 *	10.5	11.7
	**Contact area (mm^2^)**	563	604	681 *	709	684
**#3**	**Patient specific cartilage model**					
	**Peak contact pressure (MPa)**	5.7	4.8	4.5 *	6.3	7.1
	**Contact area (mm^2^)**	779	894	1013 *	947	943
	**Constant thickness cartilage model**					
	**Peak contact pressure (MPa)**	6.5	4.9	4.4 *	5.5	6.3
	**Contact area (mm^2^)**	839	958	1078 *	1029	1073
**#4**	**Patient specific cartilage model**					
	**Peak contact pressure (MPa)**	7.1	6.2 *	8.3	10.2	13.0
	**Contact area (mm^2^)**	1166	1198 *	1096	933	836
	**Constant thickness cartilage model**					
	**Peak contact pressure (MPa)**	8.1	7.2 *	7.4	8.0	8.1
	**Contact area (mm^2^)**	1101	1200 *	1151	1159	1046
**#5**	**Patient specific cartilage model**					
	**Peak contact pressure (MPa)**	5.5	5.2	4.8 *	7.7	9.1
	**Contact area (mm^2^)**	636	769 *	764	587	523
	**Constant thickness cartilage model**					
	**Peak contact pressure (MPa)**	6.3	5.2	5.0 *	6.0	7.0
	**Contact area (mm^2^)**	804	945	975 *	848	836
**#6**	**Patient specific cartilage model**					
	**Peak contact pressure (MPa)**	8.6	9.0	8.2 *	8.8	11.1
	**Contact area (mm^2^)**	466	493	517	565 *	468
	**Constant thickness cartilage model**					
	**Peak contact pressure (MPa)**	15.6	15.1	10.4	9.9 *	14.7
	**Contact area (mm^2^)**	305	375	431	457 *	369
**#7**	**Patient specific cartilage model**					
	**Peak contact pressure (MPa)**	11.3	9.8	10.0	10.0 *	15.0
	**Contact area (mm^2^)**	441	521	586	590 *	485
	**Constant thickness cartilage model**					
	**Peak contact pressure (MPa)**	11.0	7.7	5.7	5.2 *	5.9
	**Contact area (mm^2^)**	497	646	766	870 *	807
**#8**	**Patient specific cartilage model**					
	**Peak contact pressure (MPa)**	15.0	10.2	10.8	9.9 *	11.3
	**Contact area (mm^2^)**	469	514	518	530 *	505
	**Constant thickness cartilage model**					
	**Peak contact pressure (MPa)**	10.7	9.7	8.4	7.9 *	8.0
	**Contact area (mm^2^)**	398	531	584	630	661 *
**#9**	**Patient specific cartilage model**					
	**Peak contact pressure (MPa)**	10.7	9.3	9.2	7.1 *	8.5
	**Contact area (mm^2^)**	425	381	411	480 *	448
	**Constant thickness cartilage model**					
	**Peak contact pressure (MPa)**	13.0	9.4	9.1	7.7 *	8.8
	**Contact area (mm^2^)**	383	481	412	515	558 *
**#10**	**Patient specific cartilage model**					
	**Peak contact pressure (MPa)**	6.6	6.0	4.7 *	9.7	22.5
	**Contact area (mm^2^)**	802	826	951 *	750	699
	**Constant thickness cartilage model**					
	**Peak contact pressure (MPa)**	6.0	5.3	4.5 *	9.3	18.5
	**Contact area (mm^2^)**	909	990	1021 *	879	775

* represents the position with minimum peak contact pressure and maximum contact area.

## Discussion

We used a previously validated morphology-based PAO planning system [16] to perform virtual acetabular reorientation. An additional biomechanics-based module then estimated contact areas and peak contact pressures within the joint. First we used hip joint models with patient specific cartilage models and changed the LCE angle in order to increase femoral head containment and to find the optimal position with the largest contact area and lowest peak contact pressure. The same operation was then conducted with the bone models of the same hip joints by replacing the patient specific cartilage models with virtually generated constant thickness cartilage models. In the patient specific cartilage models an increase in LCE angle led to an enlarged and more homogeneously distributed contact areas and decreased peak contact pressures. Comparison of the peak contact pressures and the contact areas between the two different cartilage models showed similar results. Regression analysis quantitatively showed moderately strong correlation between both models for peak contact pressures while very strong correlation for contact areas.

In the light of our findings, several aspects need to be discussed. We did not include the acetabular labrum in our FE analysis, however the role of the labrum during load distribution is debatable in literature. While some authors promoted inclusion of the labrum [23], other authors denied the importance of its inclusion [24]. More interestingly, Henak et al. [17] showed that the labrum has a far more significant role in dysplastic hip joints biomechanics than it does in normal hips, since it supports a large percentage of the load transferred across the joint due to the eccentric loading in dysplastic hips. The same study group in a previous study [25], however, found that the labrum only supported less than 3% of the total load across the joint in normal hips. The final goal of our study was not to measure peak contact pressures and contact areas in the originally dysplastic state of our specimen, but to find an optimal position resembling a “normal” hip joint during PAO. Hence, for this purpose disregarding the labrum was acceptable.

Regarding loading conditions, a fixed body weight of 650N [21] was used, which is not patient specific. However, Zou et al. [14] justified the use of constant loading, since the relative change of contact pressure before and after PAO reorientation planning is assessed, regardless the true patient weight. Also, the applied loading conditions were derived from in vivo data from patients who underwent total hip arthroplasty (THA) [21] and thus might be just an approximation to the true loading conditions in the native joint. For simplification reasons we also did not simulate typical motion patterns such as sitting-to-standing or gait cycle. Since we only performed static loading, the conchoid shape of the hip joint, which is important, when performing dynamic loading, was also disregarded. This might be a limitation, when interpreting our results. Finally, although the CT scans were performed in the supine position and the loading condition is based on one-leg stance situation, this is not an infrequent practice [26] and previous work [27] has shown that there was no significant difference between the contact pressure in the one-leg stance reference frame and those in the supine reference frame.

Our results are reflected conclusively in the current literature. Zhao et al. [13] conducted a 3D FE analysis investigating the changes of Von Mises stress distribution in the cortical bone before and after PAO surgery. They showed the favorable stress distribution in the normal hips compared to dysplastic hips. One limitation of this study might be, that the specimens were not truly dysplastic hips. The authors created dysplasia by deforming the acetabular rim of normal hip joints. Hence, their depiction of the stress distribution in the dysplastic joint is rather an approximation. Furthermore, they used a constant thickness cartilage model. They did not estimate pressure distribution in the cartilage model but in the underlying subchondral cortical bone. Another group developed a biomechanical guiding system (BGS) [12, 26, 28]. In 2009 they presented a manuscript reporting on three-dimensional mechanical evaluation of joint contact pressure in 12 PAO patients with a 10 year follow-up. They measured radiologic angles and joint contact pressures in these patients pre- and postoperatively. The authors were able to show that after 10 year follow-up, peak contact pressures were reduced 1.7-fold and that lateral coverage increased in all patients. One limitation of their study is the use of discrete element analysis (DEA). Since the system was not only used for preoperative planning, but also as an intraoperative guidance system, the DEA represents a computationally-efficient method for modeling of cartilage stress by neglecting underlying bone stress. The cartilage models however remain largely approximated, since neither patient specific nor constant cartilage models are used, but a simplified distribution of spring elements is employed for cartilage simulation. Recently, Zou et al. [14] also developed a 3D FE simulation of the effects of PAO on contact stresses. They validated their method on 5 models generated from CT scans of dysplastic hips and used constant thickness cartilage models. The acetabulum of each model was rotated in 5° increments in the coronal plane from the original position and the relationship between contact area and pressure, as well as Von Mises stress in the cartilage were investigated, looking for the optimal position for the acetabulum. One limitation of this study is, that acetabular reorientation was roughly performed with a commercial FE analysis software (Abaqus^®^, Dassault Systèmes Simulia Corp, USA). Unlike our morphological-based planning application, their method is thus unvalidated and does not have a precise planning tool for an accurate quantification of patient specific 3D hip joint morphology.

In conclusion, our investigation contributes well to the current state of the art. First, to the best knowledge of the authors, this is the first study to use a patient specific cartilage model for biomechanics-based planning of PAO allowing for estimation of changes of contact areas and peak pressures in truly dysplastic hips. Previous studies had either investigated normal or dysplastic hips, but never the true change during virtual reorientation of the latter. Furthermore, our results seems conclusive, since the optimal position with the largest contact areas and lowest peak pressures were found within the predefined normal values [3, 29] for the investigated LCE angle. This range for safe positioning is especially important, since in real-time surgery reorientation towards the one “perfect” position might not be feasible. Finally, the comparison to constant thickness cartilage models is another novelty. Strong correlation was found for biomechanical optimization results between these two cartilage models. This is encouraging, since acquisition of patient specific cartilage requires special multiplanar arthrography imaging (e.g. CT arthrography or MRI with dGEMRIC, T1rho or T2 mapping), while constant thickness cartilage is basically always available. Although our study has its limitations and further investigation is needed, computer assisted planning with FE modeling using constant thickness cartilage might be a promising PAO planning tool providing conclusive and plausible results.
